# Salvage therapies for first relapse of SHH medulloblastoma in early childhood

**DOI:** 10.1093/neuonc/noaf092

**Published:** 2025-04-05

**Authors:** Craig Erker, Martin Mynarek, Marie Simbozel, Brandon T Craig, Virginia L Harrod, Andrea M Cappellano, Kenneth J Cohen, Vicente Santa-Maria Lopez, Andres Morales La Madrid, Chantel Cacciotti, Lorena Baroni, Ralph Salloum, Ashley S Margol, George Michaiel, Dolly Aguilera, Claire M Mazewski, Cassie N Kline, Jonathan L Finlay, Mohamed S Abdelbaki, Jeffrey C Murray, Kathleen Dorris, Bruce Crooks, Kevin F Ginn, Nisreen Amayiri, Stephan Tippelt, Gudrun Fleischhack, Svenja Tonn, Nicolas U Gerber, Alvaro Lassaletta, Jordan R Hansford, Sara Khan, Stephen W Gilheeney, Lindsey M Hoffman, Michal Zapotocky, Valérie Larouche, Shafqat Shah, Vijay Ramaswamy, Amar Gajjar, Sébastien Perreault, Sabine Mueller, Juliette Hukin, Sylvia Cheng, Zhihong J Wang, Kara Matheson, Simon Bailey, Eric Bouffet, Steven C Clifford, Giles Robinson, Christelle Dufour, Stefan Rutkowski, Lucie Lafay-Cousin

**Affiliations:** Division of Hematology/Oncology, Department of Paediatrics, IWK Health Centre and Dalhousie University, Halifax, Nova Scotia, Canada; Mildred Scheel Cancer Career Center HaTriCS4, University Medical Center Hamburg-Eppendorf, Hamburg, Germany; Department of Pediatric Hematology and Oncology, University Medical Center Hamburg-Eppendorf, Hamburg, Germany; Department of Child and Adolescent Oncology, Gustave Roussy, Paris-Saclay University, Villejuif, France; Department of Pediatrics, University of Calgary and Alberta Children’s Hospital Research Institute (ACHRI), Calgary, Alberta, Canada; Departments of Pediatric Hematology and Oncology, Dell Children’s Medical Center of Central Texas and University of Texas, Austin, Texas, USA; Division of Pediatric Oncology/BMT, Instituto de Oncologia Pediátrica-GRAACC-UNIFESP, São Paulo, Brazil; Pediatric Oncology, The Sidney Kimmel Comprehensive Cancer Center at Johns Hopkins, Baltimore, Maryland, USA; Pediatric Cancer Center Barcelona, Hospital Sant Joan de Déu, Barcelona, Spain; Pediatric Cancer Center Barcelona, Hospital Sant Joan de Déu, Barcelona, Spain; Children’s Hospital London Health Sciences, Division of Pediatric Hematology/Oncology, Western University, London, Ontario, Canada; Service of Hematology/Oncology, Hospital JP Garrahan, Buenos Aires, Argentina; Division of Hematology, Oncology and Blood and Marrow Transplant, Nationwide Children’s Hospital and The Ohio State University, Columbus, Ohio, USA; Departments of Pediatrics and Radiation Oncology, The Ohio State University College of Medicine, Columbus, Ohio, USA; Cancer and Blood Disease Institute, Children’s Hospital Los Angeles, Keck School of Medicine of University of Southern California, Los Angeles, California, USA; Division of Hematology, Oncology/Bone Marrow Transplant, Department of Pediatrics, British Columbia Children’s Hospital and University of British Columbia, Vancouver, British Columbia, Canada; Division of Hematology, Oncology/Bone Marrow Transplant, Department of Pediatrics, British Columbia Children’s Hospital and University of British Columbia, Vancouver, British Columbia, Canada; Aflac Cancer and Blood Disorder Center, Children’s Healthcare of Atlanta & Emory University, Atlanta, Georgia, USA; Division of Oncology, The Children’s Hospital of Philadelphia, Philadelphia, Pennsylvania, USA; Division of Pediatric Hematology, Oncology, and Bone Marrow Transplant, Washington University School of Medicine in St. Louis, St. Louis, Missouri, USA; Division of Pediatric Hematology/Oncology, Cook Children’s Medical Center, Fort Worth, Texas, USA; Children’s Hospital of Colorado and University of Colorado School of Medicine, Denver, Colorado, USA; Division of Hematology/Oncology, Department of Paediatrics, IWK Health Centre and Dalhousie University, Halifax, Nova Scotia, Canada; Division of Pediatric Hematology and Oncology, Children’s Mercy Hospital, Kansas City, Missouri, USA; Department of Pediatric Hematology/Oncology, King Hussein Cancer Center, Amman, Jordan; Pediatrics III, Pediatric Oncology and Hematology, University Hospital Essen, Essen, Germany; Pediatrics III, Pediatric Oncology and Hematology, University Hospital Essen, Essen, Germany; Department of Pediatric Hematology and Oncology, University Medical Center Hamburg-Eppendorf, Hamburg, Germany; Department of Oncology, University Children’s Hospital, Zurich, Switzerland; Department of Pediatric Hematology and Oncology, Hospital Infantil Universitario Niño Jesús, Madrid, Spain; Michael Rice Centre for Hematology and Oncology, The Women’s and Children’s Hospital; South Australia Health and Medical Research Institute, South Australian immunoGENomics Cancer Institute; University of Adelaide, Adelaide, Australia; Children’s Cancer Centre, The Royal Children’s Hospital, Murdoch Children’s Research Institute, University of Melbourne, Melbourne, Victoria, Australia; Division of Hematology, Oncology and Blood and Marrow Transplant, Nationwide Children’s Hospital and The Ohio State University, Columbus, Ohio, USA; Department of Pediatrics and Division of Hematology and Oncology, Mount Sinai, New York, USA; Center for Cancer and Blood Disorders, Phoenix Children’s Hospital, Phoenix, Arizona, USA; Department of Paediatric Haematology and Oncology, Second Faculty of Medicine, Charles University and University Hospital Motol, Prague, Czech Republic; Department of Pediatrics, Centre Mère-enfant Soleil du CHU de Quèbec-Université Laval, Quebec City, Quebec, Canada; The University of Texas Health Science Center, Department of Pediatric Hematology-Oncology, San Antonio, Texas, USA; Division of Haematology/Oncology, Department of Paediatrics, The Hospital for Sick Children and University of Toronto, Toronto, Ontario, Canada; Department of Oncology, St Jude Children’s Research Hospital, Memphis, Tennessee, USA; Centre Hospitalier Universitaire Sainte Justine, Université de Montreal, Montreal, Quebec, Canada; Department of Pediatrics, University of Zurich, Switzerland; Department of Neurology, Neurosurgery and Pediatrics, University of California, San Francisco, San Francisco, California, USA; Divisions of Neurology and Hematology, Oncology/Bone Marrow Transplant, Department of Pediatrics, British Columbia Children’s Hospital and University of British Columbia, Vancouver, British Columbia, Canada; Cancer and Blood Disease Institute, Children’s Hospital Los Angeles, Keck School of Medicine of University of Southern California, Los Angeles, California, USA; Division of Hematology and Oncology, Children’s Hospital of Richmond and Virginia Commonwealth University, Richmond, Virginia, USA; Research Methods Unit, Nova Scotia Health Authority, Halifax, Nova Scotia, Canada; Wolfson Childhood Cancer Research Centre, Newcastle University Centre for Cancer, Newcastle-upon-Tyne, UK; Division of Haematology/Oncology, Department of Paediatrics, The Hospital for Sick Children and University of Toronto, Toronto, Ontario, Canada; Wolfson Childhood Cancer Research Centre, Newcastle University Centre for Cancer, Newcastle-upon-Tyne, UK; Department of Oncology, St Jude Children’s Research Hospital, Memphis, Tennessee, USA; Department of Child and Adolescent Oncology, Gustave Roussy, Paris-Saclay University, Villejuif, France; Department of Pediatric Hematology and Oncology, University Medical Center Hamburg-Eppendorf, Hamburg, Germany; Section of Pediatric Hematology and Bone Marrow Transplantation, Alberta Children’s Hospital, Calgary, Alberta, Canada

**Keywords:** infant and early childhood, medulloblastoma, relapse, SHH

## Abstract

**Background:**

Sonic hedgehog (SHH) medulloblastoma is the most common molecular group of infant and early childhood medulloblastoma (iMB) and has no standard of care at relapse. This work aimed to evaluate the post-relapse survival (PRS) and explore prognostic factors of patients with nodular desmoplastic (ND) and/or SHH iMB.

**Methods:**

This international retrospective study included 147 subjects diagnosed with relapsed ND/SHH iMB between 1995 and 2017, <6 years old at original diagnosis, and treated without initial craniospinal irradiation (CSI). Univariable and multivariable Cox models with propensity score analyses were used to assess PRS for those in the curative intent cohort.

**Results:**

The 3-year PRS was 61.6% (95% confidence interval [CI], 52.2–69.6). The median age at relapse was 3.4 years (interquartile range [IQR], 2.6–4.1). Those with local relapse (40.8%) more often received salvage treatment with surgery (*P* < .001), low-dose CSI (≤24 Gy; *P* < .001), or focal radiotherapy (*P* = .008). Patients not receiving CSI (40.5%) more often received salvage marrow-ablative chemotherapy (HDC + AuHCR [*P* < .001]). On multivariable analysis, CSI was associated with improved survival (hazard ratio [HR] 0.33 [95% CI, 0.13–0.86], *P* = .04). Salvage HDC + AuHCR, while clinically important, did not reach statistical significance (HR 0.24 [95% CI, 0.0054–1.025], *P* = .065).

**Conclusions:**

Survival of patients with relapsed SHH iMB is not satisfactory and relies on treatments associated with toxicities including CSI and/or HDC + AuHCR. Cure at initial diagnosis to avoid relapse is crucial. For patients with localized relapse undergoing resection, alternative salvage regimens that avoid high-dose CSI (>24 Gy) can be considered.

**Key Points**

- The survival of relapsed SHH iMB patients is slightly above 60% and relies on treatment mainly with CSI and/or HDC + AuHCR.
- Pattern of relapse informs salvage treatment where local recurrence may be considered for treatment that avoids high-dose CSI (>24 Gy).

**Importance of the Study**

ND/SHH is the most common molecular group of iMB. Upfront HDC + AuHCR or intrathecal/intraventricular (IT/IV) methotrexate given with conventional chemotherapy has achieved improved outcomes, yet management for patients with recurrence remains poorly described. This study describes and analyzes current salvage practices, post-relapse survival, and variables that influence prognosis. This study demonstrates that salvage CSI is associated with improved survival, and although clinically meaningful, the improvement in survival with salvage HDC + AuHCR does not reach statistical significance. Patients with recurrent ND/SHH iMB remain young at the time of relapse (median age 3.4 years), highlighting the importance of maximizing cure during first-line treatment to minimize toxic salvage therapies. We also report patients with local relapses can be successfully treated with surgical resection and either non-CSI approaches or low-dose CSI (≤24 Gy) without significant differences in outcome compared to those with distant relapses who are often treated with high-dose CSI (>24 Gy).

Infant and early childhood medulloblastoma (iMB) therapy is based on the avoidance of craniospinal irradiation (CSI), often using marrow-ablative high-dose chemotherapy and autologous hematopoietic cell rescue (HDC + AuHCR) regimens or with conventional chemotherapy (CT) and intrathecal/intraventricular (IT/IV) CT to maximize survival and minimize neurocognitive late effects.^1–6^ Young patients with nodular desmoplastic (ND)/sonic hedgehog (SHH) medulloblastoma have favorable prognosis with these strategies.^5,7,8^ However, conventional CT alone results in relapse in up to 50% of patients.^9,10^

In a prior report, we described that relapsed SHH iMB has a 3-year post-relapse suvival of 60%, and show that this cohort often presents with early relapse (<12 months from diagnosis) and a high proportion of localized relapses.^11^ Although relapsed SHH iMB patients commonly received CSI-based salvage therapy, 40% underwent non-CSI salvage treatments warranting further assessment of these strategies.^11^ In the current study, we evaluate outcomes of relapsed ND and/or SHH iMB based on salvage treatment modalities, relapse patterns, and explore prognostic factors.

## Patients and Methods

### Study Population

Due to the near-complete overlap between ND histology and the SHH molecular group in iMB,^5^ the presented cohort was assembled using all patients with ND and/or SHH medulloblastoma from our previously reported cohort^11^ and 2 new cohorts from France and Germany. All patients were age <6 years at initial diagnosis^11^ with either SHH molecularly defined or ND/MBEN histology and presented with relapse following frontline CSI-sparing therapy. Patients treated with focal radiotherapy (fRT) before relapse were eligible. All patients were initially diagnosed between 1995 and 2017.

### Clinical Data Collection

Following ethics approval at participating centers, standardized forms were used to collect clinical and outcome data as previously defined and described.^11^ Data were collected as a convenience sample from patients involved in previous clinical trials (SJYC07 [NCT00602667], ACNS1221 [NCT02017964], and HIT-2000-BIS4 [NCT00303810]), patient trial registries (HIT [NCT02238899 and NCT02417324]), and individual treating centers. For patients enrolled previously in upfront clinical trials, data were collected through existing databases, and if data were not available, then data were collected through individual centers or a patient registry. Metastatic status was defined according to the Chang classification.^12^ The cohort was subsequently categorized as molecularly defined iMB SHH or ND/MBEN non-molecularly defined iMB, according to local institutional reports. Tumor histology was determined by the institutional pathologist via the patient’s pathology report, while SHH molecular classification was determined by institutionally assessed immunohistochemistry (IHC) or molecular platform (Supplementary Table 1). As methylation characterization of SHH iMB was obtained through different platforms, the data from the SHH1 and SHH β were merged, as were SHH2 and SHHγ.^13,14^ Information on constitutional genetic predisposition was not collected. CSI dosing was dichotomized to reflect the bimodal dose distribution seen in clinical practice and in our cohort. We used 24 Gy as a cutoff, with ≤24 Gy being called low-dose CSI and >24 Gy being called higher-dose CSI, to reflect clinical practice and historical data that doses of 23.4 Gy result in less severe neuropsychological toxicity than higher CSI doses.^6,15^

### Statistical Analysis

Non-parametric analyses were used to assess for differences between the ND non-molecularly defined group and SHH-defined cohorts. Post-relapse survival (PRS), defined as the time from the first relapse to death or last follow-up, was examined using patients in the curative intent cohort. PRS was performed using Kaplan–Meier analysis and significance testing (α = 0.05) based on log-rank testing.

Univariable and multivariable Cox proportional-hazards models were used to estimate the hazard ratio (HR) to death. The Kaplan–Meier curves and proportional-hazards models were adjusted using an inverse probability of treatment weight. The propensity score weight was stabilized using standardized mean weight to compensate for imbalances in treatment prior to relapse.^16^ The propensity score weight was generated by fitting a logistic regression model with CSI treatment as the outcome, adjusting for confounders believed to be related to CSI treatment after relapse and the study outcome, mortality. Variables included in the propensity score model included diagnosis era, molecular subgroup, frontline fRT, age at relapse, pattern of relapse, upfront CT type (CT alone vs HDC + AuHCR), time from diagnosis to relapse, and sex. Salvage CSI and HDC + AuHCR variables were assessed as time-dependent variables accounting for the time from initial relapse until a patient received CSI and/or HDC + AuHCR to account for the possibility of immortal time bias. Variables for the multivariable analysis were chosen post hoc, based on clinical relevance and/or univariable survival analysis results (*P* < .1). SAS 9.4 (Cary, NC) was used for all statistical analyses.

## Results

### Patient Characteristics and Treatments Prior to Relapse

A total of 147 patients were included in the study, 129 received curative intent salvage therapy while 18 underwent palliative treatment. Forty-one were not previously reported.^11^ One hundred and thirteen of the 147 patients had molecularly defined SHH MB with the remainder having ND/MBEN non-molecularly defined iMB. Fifty percent (*n* = 74) were originally enrolled in prospective clinical trials for upfront disease and suffered relapse or were part of registries: 19 from SJYC07, 10 from ACNS1221, 30 from the HIT trials and registry, and 15 from the United Kingdom Children’s Cancer Leukemia Group (UK-CCLG). The remaining patients were collected from 20 individual international institutions.

The patient characteristics and treatment modalities at initial diagnosis are described in Table 1. The median age at initial diagnosis of MB was 27 months (range 1–70 months). Localized disease was present in 74.3%. Gross total resection (GTR) of the primary mass was achieved in 72.8%. Regarding the 113 (76.9%) patients with molecularly defined SHH MB, 13 (11.5%) had non-ND/MBEN histology (9 classic iMB and 4 large-cell anaplastic iMB). Methylation subtyping was available in 37.2% of the SHH MB cohort, segregating into SHH1/β and SHH2/γ for 61.9% and 38.1%, respectively (Supplementary Table 2).

**Table 1.** Description of curative intent, palliative intent, and overall cohort

	Curative intent (*n* = 129)	Palliative intent (*n* = 18)	Overall cohort (*n* = 147)
**U** **PFRONT**			
Gender (male)	78 (61%)	8 (44%)	86 (59%)
Diagnosis era 1995–2006 2007–2017	38 (30%) 91 (70%)	5 (28%) 13 (72%)	43 (29%) 104 (71%)
Age at diagnosis <24 months	49 (38%)	12 (67%)	61 (42%)
Metastatic status M0	96 (76%)	11 (61%)	107 (74%)
Histology Classic ND/MBEN LCA	9 (7%) 117 (91%) 3 (2%)	0 17 (94%) 1 (6%)	9 (6%) 134 (91%) 4 (3%)
Molecular subgroup SHH1/β SHH2/γ	22 (60%) 15 (40%)	4 (80%) 1 (20%)	26 (62%) 16 (38%)
HDC (yes)	24 (19%)	2 (11%)	26 (18%)
IT/IV chemotherapy	34 (26%)	4 (25%)	38 (26%)
Focal RT	6 (5%)	2 (11%)	8 (5%)
**R** **ELAPSE**			
Relapse timepoint On therapy Off therapy	45 (35%) 79 (61%)	11 (65%) 6 (35%)	56 (38%) 85 (58%)
Time from diagnosis to relapse <12 months	49 (38%)	9 (50%)	58 (40%)
Detection of relapse Routine imaging or CSF Symptomatic relapse	86 (67%) 17 (13%)	6 (43%) 8 (57%)	92 (63%) 25 (17%)
Relapse pattern Local Disseminated ± local	57 (44%) 70 (54%)	3 (18%) 14 (82%)	60 (41%) 84 (57%)
Surgery (yes)	65 (50%)	3 (17%)	68 (46%)
HDC (yes)	36 (28%)	0	36 (25%)
Alive (yes)	76 (59%)	1 (6%)	77 (52%)

Abbreviations: ND/MBEN, nodular desmoplastic/medulloblastoma with extensive nodularity; LCA, large-cell/anaplastic; HDC, high-dose chemotherapy; IT/IV, intrathecal/intraventricular; RT, radiation therapy; CSF, cerebrospinal fluid.

After initial surgical resection, patients primarily received adjuvant CT. The most commonly used regimens were the HIT-SKK regimen^17^ followed by Head Start induction treatment^8^ in 39.2% and 20%, respectively (Supplementary Figure 1). IT/IV CT was administered in 26.2% of the cases. Twenty-one patients (14.3%) underwent maintenance therapy, with 12 (57.1%) receiving maintenance according to the SJYC07 protocol.^9^ HDC + AUHCR strategies were used in 17.7%, predominantly with 1 cycle (69.2%) or 3 sequential cycles (30.8%). Only 8 patients (5.4%) received fRT. At the completion of initial therapy, 51% achieved complete response, 7.5% incomplete response, 36.7% progressive disease, and 4.8% had unknown response status.

### Pattern of Relapse

**Figure 1.**
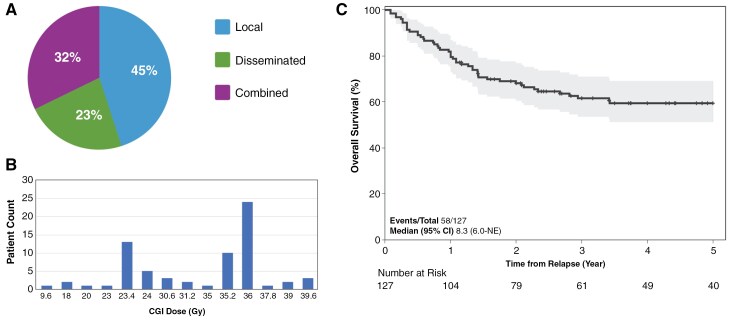
(A) Pie chart of pattern of relapse for curative intent cohort; (B) bar graph for salvage CSI dosing for curative intent cohort; (C) Kaplan–Meier plot showing the overall survival of curative intent cohort with the gray zone representing the 95% confidence interval.

The median time to relapse/progression from initial diagnosis was 14.7 months (interquartile range [IQR] 9.0–18.4) or 4 months (IQR 0–9) from treatment completion. Relapse on therapy occurred in 38.1%, while only 4 patients relapsed beyond 30 months from diagnosis (31, 41, 56, and 140 months). The median age at the time of relapse was 3.4 years (IQR 2.6–4.1). Only 17.0% of the patients presented with symptomatic relapse, whereas the majority were detected during routine surveillance. Local relapse accounted for 40.8%, while combined or disseminated relapse was reported in 57.1% (unknown 2%; Figure 1A). Patients with localized relapse were more likely than those with combined or disseminated relapse to receive salvage regimens that involved low dose CSI dose ≤24 Gy (*P* < .0001), focal irradiation, and undergo surgical resection (*P* < .0001).

### Salvage Therapy for the Curative Intent Cohort

The 18 patients who underwent palliative management were younger at the time of diagnosis (*P* = .02) and at relapse (*P* = .045) and more likely to present with clinically symptomatic (*P* = .003) and disseminated relapse (*P* = .03).

Of the 129 patients treated with curative intent, 99 (76.7%) were molecularly defined SHH and 30 (23.3%) were histologically defined ND iMB. Of the 99 patients with molecularly defined SHH, 92% were diagnosed via various molecular platforms(Supplementary Table 1) with DNA methylation being the most common (*n* = 71) and the remaining 8% diagnosed by IHC. The characteristics of patients with molecularly defined SHH iMB and histologically defined ND iMB were not significantly different (Table 2). Given the similarity, the 2 cohorts were combined (*n* = 129) to describe salvage modalities and outcomes for the curative intent cohort.

**Table 2.** Curative intent cohort comparing molecularly SHH iMB and ND non-molecularly defined iMB

	SHH (*n* = 99)	ND (*n* = 30)	*P*
**UPFRONT**			
Gender (male)	63 (63%)	15 (50%)	.18
Diagnosis era 1995–2006 2007–2017	25 (25%) 74 (75%)	13 (43%) 17 (57%)	.06
Age original diagnosis <24 months	37 (37%)	12 (40%)	.80
Metastatic status M0 M+	72 (75%) 24 (25%)	24 (80%) 6 (20%)	.57
Histology Classic ND/MBEN LCA	9 (9%) 87 (88%) 3 (3%)	0 30 (100%) 0	.13
HDC (yes)	15 (15%)	9 (30%)	.07
**RELAPSE**			
Relapse timepoint On therapy Off therapy	37 (39%) 57 (61%)	8 (27%) 22 (73%)	.21
Time diagnosis to relapse <12 months	40 (40%)	9 (30%)	.30
Relapse pattern Local Disseminated ± local	47 (48%) 50 (51%)	10 (33%) 20 (67%)	.25
Surgery (yes)	52 (53%)	13 (43%)	.14
Radiation type No RT CSI Focal RT only Systemic radioisotope	28 (28%) 55 (56%) 15 (15%) 1 (1%)	5 (17%) 23 (77%) 1 (3%) 1 (3%)	.10
HDC (yes)	29 (29%)	7 (23%)	.52
Relapse treatment combo CSI alone CSI + conventional chemo CSI + HDC HDC without CSI Non-HDC only Other non-CSI approach	16 (16%) 30 (30%) 20 (20%) 9 (9%) 21 (21%) 3 (3%)	9 (30%) 10 (33%) 3 (10%) 4 (13%) 4 (13%) 0	.33
Alive (yes)	59 (60%)	17 (57%)	.78

Abbreviations: SHH, sonic hedgehog; ND, nodular desmoplastic; ND/MBEN, nodular desmoplastic/medulloblastoma with extensive nodularity; LCA, large-cell/anaplastic; HDC, high-dose chemotherapy; RT, radiation therapy; CSI, craniospinal irradiation; combo, combination; chemo, chemotherapy.

#### Surgery

Half of the patients underwent surgical resection at the time of relapse. Among them, 63.3% achieved GTR. Surgery was more likely to be attempted for those with local relapse than in metastatic dissemination, 74.6% versus 36.7%, respectively (*P* < .0001).

#### RT

RT was a core modality of salvage, used in 96 patients (74.4%). Salvage RT was given most often as CSI in 78 patients (81.3%). CSI dose was available in 69 (88.5%) patients, with the median dose and boost delivered being 35.2 Gy [IQR 23.4–36] and 54.9 Gy [54–55.9], respectively. Thirty-two percent of the patients received CSI without CT [median dose 36.0 Gy, IQR 31.7–36.0]. Twenty-three (33.3%) patients received low-dose CSI (≤24 Gy; Figure 1B). Patients with localized relapses were more likely to receive low-dose salvage CSI (*P* < .001). Age at relapse was not statistically different (*P* = .58) for those receiving ≤24 Gy compared to those who received higher-dose CSI (>24 Gy). For the 30 patients with local relapse receiving CSI, the median dose was 23.4 Gy (IQR 23.4–36 Gy; Supplementary Figure 2).

Children who received salvage CSI were older at the time of relapse than those who did not (44.4 months vs 34.8 months; *P* < .0001) and were more likely to have later relapse from initial diagnosis (15.6 months vs 11 months; *P* = .006). Children who received salvage CSI were less likely to have received HDC + AuHCR as part of their salvage therapy compared to children who did not receive salvage CSI (16.7% vs 45.1%; *P* = .0004).

Of the 18 children receiving non-CSI-based salvage irradiation, 16 underwent salvage fRT at a median dose of 54 Gy [IQR 50.4–54], and the 2 remaining patients received systemic radioisotope. Thirteen of 16 (81.3%) patients who received salvage fRT had a localized relapse.

#### CT and targeted treatments

CT was given as salvage to 76.9% of patients. Of all patients receiving CT, 47.2% received conventional CT, 36.2% used HDC + AuHCR, and the remainder received maintenance CT alone. Patients who underwent HDC + AuHCR predominantly received 1 cycle of consolidation (47.2%), most commonly with carboplatin, etoposide, and thiotepa (58.8%). Sequential consolidation with 3 cycles was reported in 33.3%, mainly using carboplatin and thiotepa (83.3%). Other combinations of agents, including busulfan/thiotepa and melphalan/carboplatin, among others, accounted for the remaining third of the marrow-ablative regimens. None of the children who underwent salvage HDC + AuHCR had previously received HDC + AuHCR at initial diagnosis. Twenty patients also received IT/IV CT as a part of salvage therapy. Eleven of them previously had IT/IV during upfront treatment. Eleven patients (8.5%) received SHH inhibitors during salvage treatment (8 patients received vismodegib and 3 sonidegib).

#### Treatment combinations

For the 78 patients who underwent salvage CSI, most also received either CT (51.3%) or HDC + AuHCR (16.7%). In those receiving CT in combination with CSI, CT was used pre-, post-, or both pre- and post-RT in 31.6%, 42.1%, and 26.3%, respectively. The median dose of CSI used in combination with CT or HDC + AuHCR was not significantly different compared to CSI alone (*P* = .09). Patients who received salvage CSI ≤24 Gy (82.6% (*n* = 19) vs 37.2% (*n* = 16)) were more likely to have undergone salvage surgery (*P* = .0004).

Thirteen of the 36 patients who received HDC + AuHCR also underwent CSI. Five of 23 patients treated with CSI ≤24 Gy also underwent salvage HDC. In all cases, the CSI followed HDC + AuHCR at a median time of 1 month (range 0–14 months). For those who received both salvage HDC + AuHCR and CSI, there was no difference in the frequency of low-dose versus higher-dose CSI (*P* = .5005). The remaining 23 patients of the 36 who recevied HDC + AuHCR did not receive CSI after HDC + AuHCR, although 7 received fRT and 2 had systemic radioisotope.

Overall, 72 patients (55.8%) either received no RT (*n* = 33) or underwent CSI at a dose ≤24 Gy (*n* = 23) or fRT only (*n* = 16). Treatment combinations for the patients who received CSI ≤24 Gy are illustrated in Supplementary Figure 3. Of the 33 patients who did not receive any RT, 14 (42.4%) underwent surgery with additional CT (7 HDC + AuHCR and 7 conventional CT). The remaining 19 patients were treated without surgery and received HDC + AuHDC (36.8%), CT (42.1%), and IT/IV CT (15.7%), with 1 patient missing CT details. Patients treated without RT were more likely to receive salvage HDC than those treated with RT. Fourteen (42.4%) patients treated without RT were being treated for local relapse.

### Causes of Death

Forty-two patients (33.1%) died of disease, and 11 (8.7%) died of other causes, including 3 from acute treatment toxicity (1 sepsis during HDC + AuHDC with thiotepa, etoposide, and carboplatin; 1 hemorrhage with conventional CT; and 1 unknown), 2 from late treatment-related complications (irradiation-related chronic lung disease and acute subdural hemorrhage), 5 (3.9%) from subsequent malignancies (including 1 hip osteosarcoma, 1 bithalamic glioma, 1 glioblastoma, 1 leukemia, and 1 unknown subsequent malignancy), and 1 of unknown cause. Of the 5 patients who died of subsequent malignancy, all had ND/MBEN histology and were <3 years of age at original diagnosis (range 0–2.8 years). The onset of subsequent malignancy from relapse was at a mean of 8.9 years (range 7.2–12.5).

### PRS and Associated Prognostic Factors

**Figure 2.**
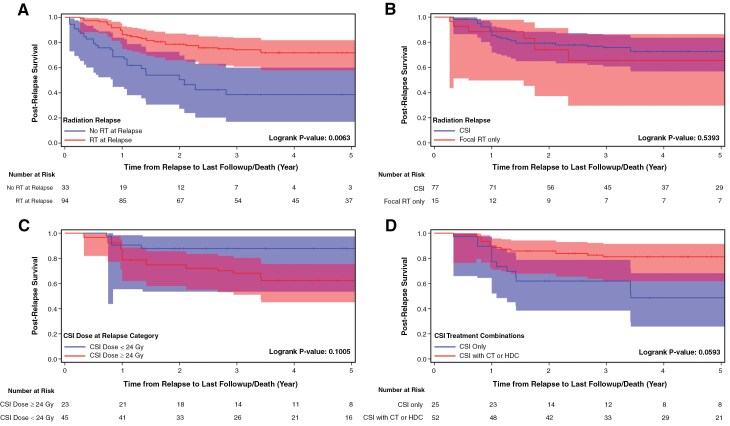
Kaplan–Meier plot showing post-relapse survival for curative intent cohort with the 95% confidence intervals demonstrated for (A) those treated with and without salvage radiation therapy; (B) treatment with salvage CSI compared to those treated with salvage focal radiation therapy; (C) treatment with salvage CSI at ≤24 Gy compared to >24 Gy; (D) treatment with salvage CSI alone compared to salvage CSI with systemic chemotherapy.

At a median follow-up of 32.3 months (IQR 14–72), the 3-year PRS was 61.6% (95% CI, 52.2–69.6; Figure 1C). Patients treated with salvage RT (CSI or fRT) had a 3-year PRS of 74.8% (95% CI, 60.7%–83.6%) compared to 38.5% (16.9%–59.9%) for those who did not (*P* = .006; Figure 2A). However, no statistical difference was detected when comparing CSI with fRT only (*P* = .53; Figure 2B). Of interest, the 3-year PRS for the 23 patients who received salvage CSI ≤24 Gy was 87.8% (95% CI, 53.5, 97.3) compared with 68.2% (95% CI, 51.1, 80.4) for those with CSI >24 Gy (*P* = .1005; Figure 2C). Similarly, the 3-year PRS for those who received fRT was 65.6% (29.6–86.4). The PRS for patients treated with CSI in combination with CT or HDC + AuHCR as compared to salvage CSI only (81.2% [61.8–91.3] vs 62.0% [38.4–78.7], *P* = .059; Figure 2D). PRS for patients with localized relapse versus those with disseminated relapse was not statistically different (*P* = .35).

In univariate Cox proportional-hazards analysis, younger age at relapse (<36 months old) and early relapse (<12 months from diagnosis) were associated with worse PRS, while the use of salvage CSI was associated with better PRS (Table 3). Although salvage HDC + AuHCR and surgery at relapse were not significant in improving PRS (*P* = .083 and .06, respectively), they were included in multivariable testing. In a post hoc analysis, there was a statistically significant interaction between salvage HDC + AuHCR without CSI versus salvage CSI without HDC + AuHCR with a crossing of survival curves. Patients who received both CSI and HDC + AuHCR appeared to do best (Supplementary Figure 4), while patients treated without either CSI or HDC + AuHCR had the lowest 3-year PRS at 31.6% (95% CI, 10.7%–55.3%). The upfront metastatic status, the use of HDC + AuHCR or CT at initial diagnosis, the initial histology subtype, and the pattern of relapse were not associated with PRS. The use of upfront fRT also did not impact PRS (*P* = .18), although only 6 patients had upfront fRT.

**Table 3.** Univariate analysis for PRS for the curative intent cohort

Variable	3-year PRS (95% CI)	Overall log-rank *P*	Point estimate HR (95% CI)	HR *P*
**UPFRONT**				
Sub-cohort SHH (ref) ND	62.7 (48–73) 68.3 (42–84)	.87	0.94 (0.49–1.80)	.84
Metastatic status M0 (ref) M+	68.5 (54–79) 43.2 (23–62)	.18	1.56 (0.86–2.83)	.14
Upfront HDC No HDC (ref) HDC	61.3 (48–72) 77.8 (45–92)	.40	0.62 (0.23–1.68)	.34
Upfront focal RT Yes No (ref)	26.6 (3–62) 65.4 (53–75)	.18	2.07 (0.75–5.71)	.16
Upfront IT/IV therapy Yes No (ref)	56.8 (35–74) 65.9 (51–77)	.69	1.15 (0.65–2.06)	.63
**RELAPSE**				
Time, diagnosis to relapse ≥12 months (ref) <12 months	73.1 (58–83) 48.3 (28–65)	.007	2.67 (1.52–4.67)	.0006
Age at relapse ≥36 months <36 months (ref)	70.7 (57–81) 50.6 (27–70)	.056	0.48 (0.28–0.83)	.009
Pattern of relapse Local (ref) Disseminated ± local	70.5 (51–83) 58.8 (43–71)	.35	1.40 (0.80–2.46)	.24
Surgery No Yes (ref)	56.7 (37–72) 73.7 (58–84)	.13	1.71 (0.97–3.02)	.06
CSI (time-dependent) Yes vs No	N/A		0.54 (0.30–0.96)	.037
HDC (time-dependent) Yes No (ref)	N/A		0.55 (0.28–1.08)	.08
Relapse therapy combinations CSI + HDC CSI + non-HDC CSI alone HDC ± non-HDC Non-HDC only Other therapy	95.3 (34–100) 76.2 (51–90) 62.0 (38–79) 69.2 (39–87) 27.2 (8–51) 55.4 (1–93)	.0034	N/A	

Abbreviations: PRS, post-relapse survival; HR, hazard ratio; SHH, sonic hedgehog; ND, nodular desmoplastic; HDC, high-dose chemotherapy RT, radiation therapy; IT/IV, intrathecal/intraventricular; CSI, craniospinal irradiation; N/A, not applicable.

**Figure 3.**
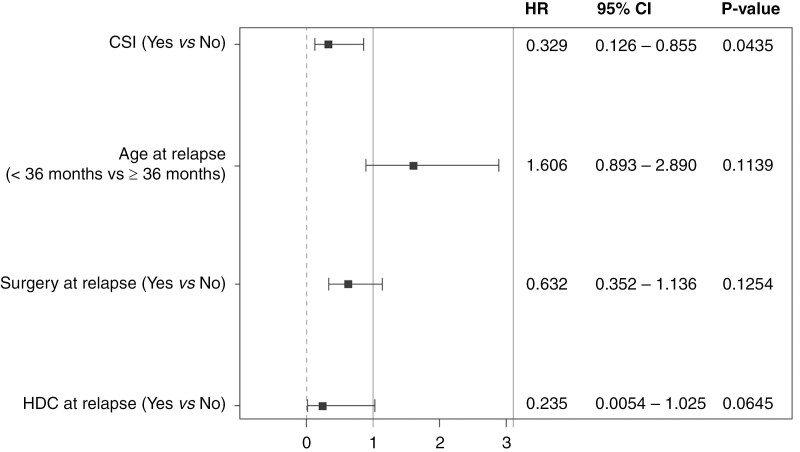
Post-relapse survival forest plot of exploratory multivariable analysis of curative intent cohort. The whiskers represent the 95% confidence interval of the hazard ratio (HR), which is represented by the small square.

Multivariable analysis with propensity scoring analyzed the use of surgery at relapse, age at relapse, CSI (time-dependent), and salvage HDC + AuHCR (time-dependent). The median time to CSI receipt from relapse was 2.5 months (IQR 1.0–6.0 months), and the median time to HDC + AuHCR was 3.0 months (IQR 1.0–4.0 months). An interaction term (HDC + AuHCR + CSI) was placed in the model to adjust for the relationship between HDC + AuHCR and CSI. Time from diagnosis to relapse was highly correlated with age at relapse and therefore was not included in the model due to multicollinearity. The age at relapse (<36 vs ≥36 months) was deemed more clinically significant and so was preferentially included in the model. A second model was run adding in the molecularly defined versus non-molecularly defined MB, which did not influence the model outcome. On the final multivariable model (*n* = 117), CSI was a significant predictor of PRS with an HR of 0.33 (95% CI, 0.13–0.86, *P* = .044) while HDC + AuHCR at relapse showed an HR of 0.24 (95% CI, 0.005–1.03, *P* = .065; Figure 3).

## Discussion

We show that many patients with relapsed SHH medulloblastoma can be retrieved with a 3-year PRS of 61.6% (95% CI, 52.2–69.6). We also corroborate other reports indicating that 40% of patients with relapsed SHH/ND iMB treated without upfront CSI are localized.^9–11^ Similar to patients with other iMB subgroups, salvage therapy for SHH/ND iMB relies heavily on RT, used in 74.4% of the patients. Salvage CSI was associated with significant survival benefits in both univariate and multivariable analyses. Previous studies showed the role of salvage CSI as a potentially successful strategy in CSI-naive relapsed iMB.^18–20^ However, unique to our cohort are the characteristics of various salvage strategies employed, including over half of the cohort (55.8%) that received either no RT (25.6%), fRT only (12.4%), or low-dose CSI (17.8%). Although the PRS for patients who received RT was better than those who did not, no significant difference in PRS was detected between salvage CSI and salvage fRT, possibly related to small fRT numbers and their predominance of local relapse.

The median dose of salvage CSI delivered for the entire cohort was 35.2 Gy, which is not unexpected as a dose of 36 Gy CSI is a well-accepted dose for upfront therapy in older children with high-risk MB. Importantly, our cohort had CSI doses that were largely dichotomized and did not include any patients with salvage therapy between >24 Gy and <30.6 Gy, making assessment of this intermediate dose range unfeasible. We previously reported a tendency for some patients with relapsed SHH/ND iMB to receive salvage therapy without CSI.^11^ Here we describe one-third of patients who received salvage CSI did so with low-dose CSI (≤24 Gy). Similar to those treated with salvage fRT, patients who underwent low-dose CSI presented more frequently with local relapse (78%). Our data show that physicians are managing patients with local relapse differently compared to patients with disseminated or combined relapse. Patients with localized disease were more likely to undergo disease resection at relapse and/or to receive fRT or low-dose CSI, suggesting a bias in the allocation of irradiation modalities according to the pattern of relapse. With this allocation of low-dose CSI preferentially to those with localized relapse, there were only 4 events reported for the 23 patients who received CSI ≤24 Gy. With the limitation of the retrospective nature of this cohort, our data suggest that some patients with localized relapsed SHH/ND iMB can be successfully salvaged with strategies that avoid high-dose CSI, principally when salvage surgical resection is undertaken. This finding also highlights the importance of obtaining a state of minimal gross residual prior to radiation or CT for those with localized relapse.

We show that PRS for patients salvaged with CT and CSI, compared to those who received CSI alone, survival was 81.2% versus 62.0%, and although not statistically significant (*P* = .059) is an area deserving further investigation. The median dose of CSI was not statistically different when used alone or in conjunction with conventional CT or HDC + AuHCR. Whether salvage CT can safely allow for lower doses of CSI for relapsed SHH/ND iMB is also worth additional study. Our data caution against the use of a CSI-alone approach in children with relapsed SHH iMB. Although the timing of CSI after relapse was accounted for through time-dependent analyses, the relevance of the precise timing of salvage conventional CT, whether before or after salvage CSI, remains unknown.

The use of salvage HDC + AuHCR was not statistically significant in univariate (HR 0.55 [0.28–1.08], *P* = .08) or multivariable analyses (HR 0.24 [95% CI, 0.0054–1.025], *P* = .06). However, these results with salvage HDC + AuHCR appear clinically meaningful as patients who received salvage HDC + AuHCR without CSI fared better than those who received neither CSI nor HDC + AuHCR (*P* = .03). Ultimately, salvage CSI outperformed HDC in multivariate modeling. It is important to recognize the limitation of this model even with propensity scoring, as these patients were not randomly allocated and the number of patients receiving HDC + AuHCR (*n* = 36) was smaller compared to that of CSI (*n* = 78). There were no patients who underwent HDC + AuHCR upfront and then again at salvage, which questions the applicability of such an approach, although further investigation may be needed.^8–10,21^ We suggest that for patients treated with conventional CT as used in the HIT-2000 or as currently investigated in the SJiMB21 clinical trial (NCI-2022-07099), salvage HDC + AuHCR with or without low-dose CSI represents an important salvage strategy.^5,22^ However, if CSI is given as part of salvage with HDC + AuHCR, the current practice has been to give CSI after completion of HDC + AuHCR as a consolidation approach. This is especially important if there is consideration of methotrexate as part of the treatment regimen, which should be given prior to CSI to avoid increased risk of leukoencephalopathy. While international collaboration is underway through SIOPE and the CONNECT consortium to compare upfront treatment modalities for SHH/ND iMB, a large international effort will also be needed to assess patients who will fail these strategies.

These children remain young at the time of relapse and therefore vulnerable to treatment-related toxicities, raising the importance of considering the associated harms of salvage therapies and highlighting the priority of achieving an upfront cure. Likely underestimated due to the lack of data completeness, we report that nearly one-third of these young patients required hearing support. Additional analyses of long-term toxicities such as neurocognitive and ototoxicity data for these patients are needed to assess the post-relapse intellectual profile of survivors.

At a median follow-up of 32.3 months from relapse, subsequent malignancies were reported in 5 (3.9%) patients. The cumulative incidence of subsequent malignancies in older children treated for MB with CSI and CT has been estimated at 4.2% (1.9%–6.5%) at 10 years.^23^ The true frequency of second malignancy in our cohort is likely underestimated, as only those leading to death were captured, but the number we report is sizeable. RT’s impact on patients with SHH iMB is important to consider, as young children with SHH have a high rate of underlying germline mutation (up to 20%), notably *SUFU* and *PTCH1* mutation, predisposing them to multiple cancers and may be further exacerbated by RT.^24,25^ Therefore, the pros and cons of omitting CSI in this population should be considered, especially if HDC + AuHCR is a salvage option. Interestingly, none of the described subsequent malignancies in our cohort were ones commonly associated with *SUFU* or *PTCH1* germline alterations.^24^ If not undertaken at initial diagnosis, a genetic referral at the time of relapse is critical to integrate the risk of basal cell carcinoma or meningioma associated with Gorlin syndrome and radiation exposure.^26–28^

This study combined patients originally enrolled in clinical trials prior to relapse and those from national and institutional databases. Combining these groups of clinical data will result in added data heterogeneity, leading to variability in data completeness and accuracy, increasing caution in the application of our results. Also, our cohort of relapsed SHH MB did not undergo a central review of imaging. It is possible that some patients with relapsed disease may have had a second de novo tumor rather than relapsed SHH iMB which may be important to consider given the risk of constitutional genetic predisposition in these patients.

A significant limitation of this work is the lack of constitutional genetic data. Specifically, in our cohort, information on germline *ELP1* mutation was not collected, limited by the retrospective nature of the study. Its occurrence should be low given the median age of this *ELP1* germline mutation is 7.3 years old and is restricted to the SHH3/α subgroup.^29,30^ Also, we did not capture the prevalence of TP53 mutations in our patient population, which is known to be a poor predictor in older children with SHH medulloblastoma mainly 8–17 years old, generally clustering with SHH3, although testing is recommended in SHH MB for those with LCA (large-cell/anaplastic) histology who are 4 or more years old.^31,32^ Eleven percent of our patients were aged 4 or above at diagnosis, although none of these had LCA histology. Otherwise, TP53 mutation is not prognostic in SHH1/β and SHH2/γ subgroups which given the age of our cohort would be the vast majority.^33^ Similarly, we did not capture *MYCN* or *GLI* amplification in our cohort. However, again it is the SHH3/α subgroup that is enriched for this alteration, and its presence is not prognostic in SHH1/β and SHH2/γ subgroups.^33,34^ Due to the limitations of available biology details, we are not able to exclude the possibility of a rare patient with an SHH3/α subgroup iMB in our cohort.

Heterogenous methods were used to confirm the diagnosis of the SHH MB subgroup, which limits data uniformity stemming from the use of retrospective data. Some methods are less precise than others, which may give variability in misclassification depending on the platform used. There were also a limited number of patients with SHH1/β and SHH2/γ methylation subgroup status (28%) which prevented us from assessing the possible impact of methylation subgrouping and salvage outcomes. The increasing integration of methylation status with copy number variations in the most recent clinical trials may contribute to delineating varying risk groups for relapsed SHH iMB.^22,35^

The comprehensive description of this large cohort of patients with relapsed SHH/ND iMB provides useful information for parents and treating physicians for counseling at the time of relapse. Importantly, successful salvage is not guaranteed, and CSI, the modality initially desired to be avoided, is often the best chance of cure after relapse. This emphasizes the need to maximize upfront cure and to be highly cautious when attempting to reduce therapies with proven efficacy. The pattern of relapse, prior therapies, and age at relapse should guide salvage management. Further efforts to characterize tumor biology in this cohort of patients may further help stratify SHH iMB in the relapse setting. Therapies that avoid high-dose salvage CSI may be considered for patients with localized relapse after surgical resection. Further characterization of these patients will benefit from prospective international collaboration.

## Supplementary Material

noaf092_suppl_Supplementary_Tables_S1-S2_Figures_S1-S4

## Data Availability

Data via statistical analysis will be made available upon reasonable request to the corresponding author.
