# Supplementary material for: Salvage therapies for first relapse of SHH medulloblastoma in early childhood
Source: Neuro Oncol. 2025 Apr 5;27(8):2158–69. doi: 10.1093/neuonc/noaf092 (PMC12448823; doi:10.1093/neuonc/noaf092)
Supplement: noaf092_suppl_Supplementary_Tables_S1-S2_Figures_S1-S4 [file noaf092_suppl_supplementary_tables_s1-s2_figures_s1-s4.zip › Supplemental Figure 2-Edit.pptx]

## Slide 1
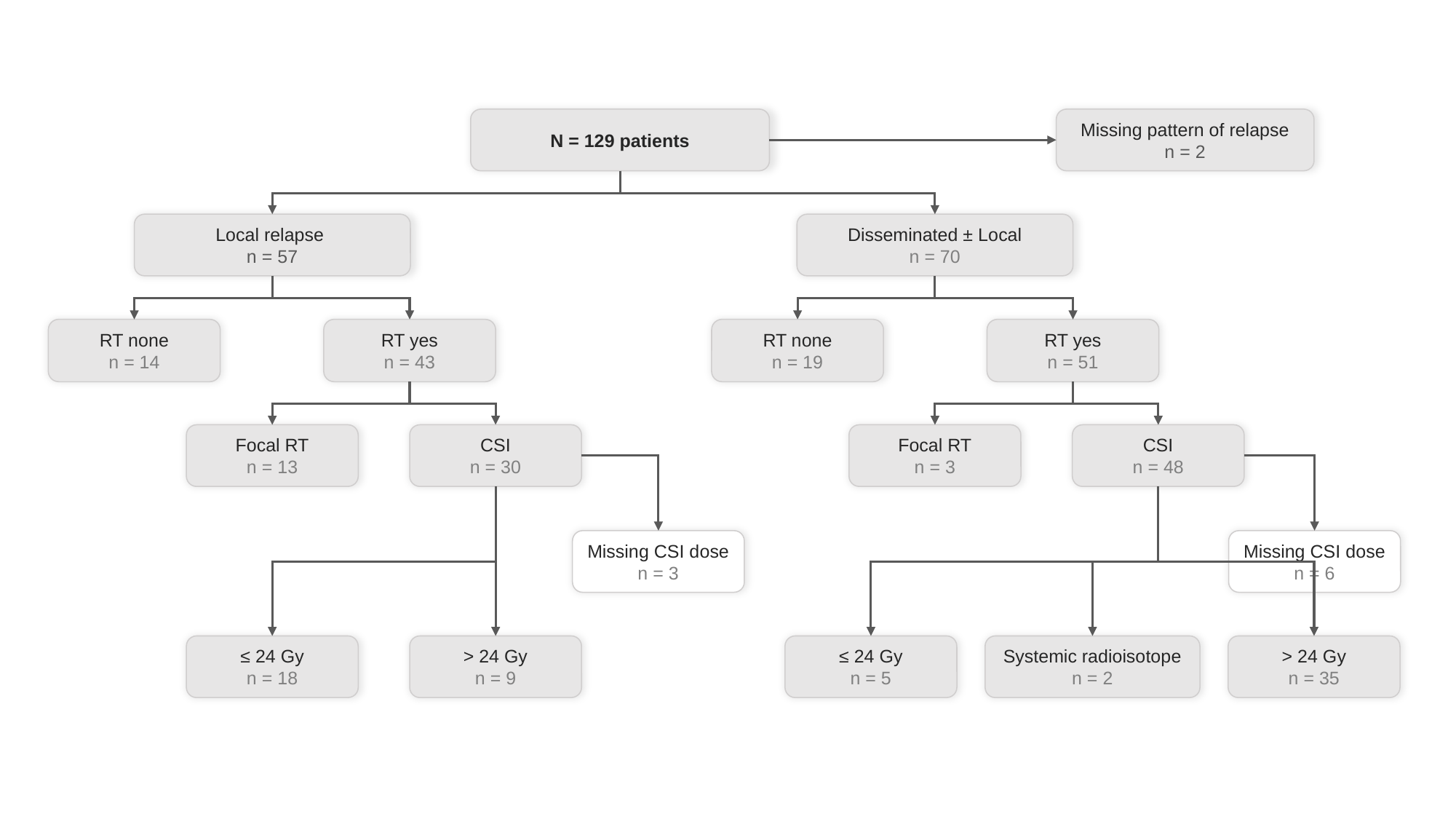

N = 129 patients
Missing pattern of relapse
n = 2
Local relapse
n = 57
Disseminated ± Local
n = 70
RT none
n = 14
RT yes
n = 43
RT none
n = 19
RT yes
n = 51
Focal RT
n = 13
CSI
n = 30
Focal RT
n = 3
CSI
n = 48
Missing CSI dose
n = 3
Missing CSI dose
n = 6
≤ 24 Gy
n = 18
> 24 Gy
n = 9
≤ 24 Gy
n = 5
Systemic radioisotope
n = 2
> 24 Gy
n = 35
