# Supplementary material for: Salvage therapies for first relapse of SHH medulloblastoma in early childhood
Source: Neuro Oncol. 2025 Apr 5;27(8):2158–69. doi: 10.1093/neuonc/noaf092 (PMC12448823; doi:10.1093/neuonc/noaf092)
Supplement: noaf092_suppl_Supplementary_Tables_S1-S2_Figures_S1-S4 [file noaf092_suppl_supplementary_tables_s1-s2_figures_s1-s4.zip › Supplemental Figure 3-Edit.pptx]

## Slide 1
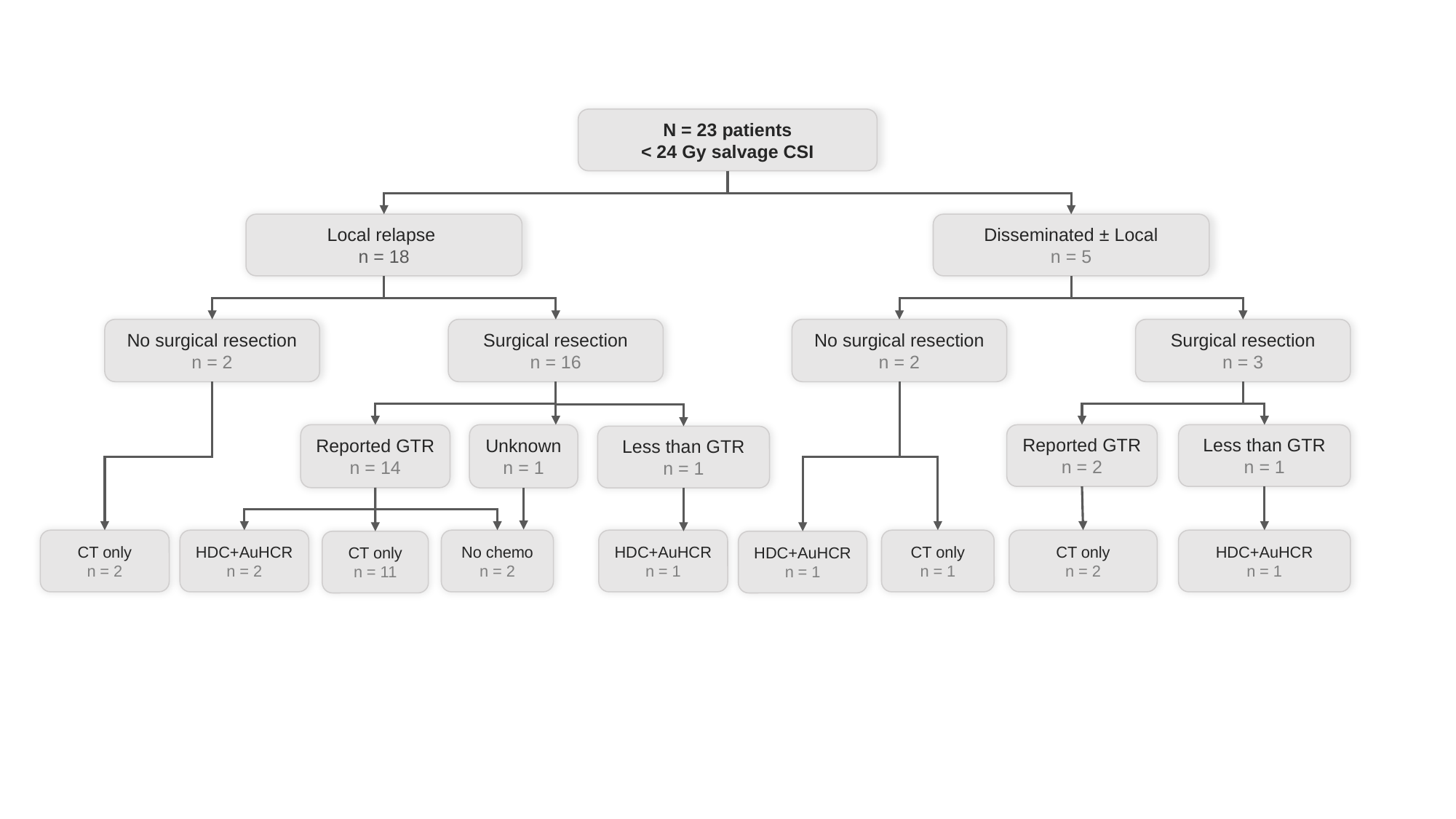

N = 23 patients
< 24 Gy salvage CSI
Local relapse
n = 18
Disseminated ± Local
n = 5
No surgical resection
n = 2
Surgical resection
n = 16
No surgical resection
n = 2
Surgical resection
n = 3
Unknown
n = 1
Reported GTR
n = 14
Reported GTR
n = 2
Less than GTR
n = 1
Less than GTR
n = 1
CT only
n = 2
HDC+AuHCR
n = 2
No chemo
n = 2
HDC+AuHCR
n = 1
CT only
n = 1
CT only
n = 2
HDC+AuHCR
n = 1
CT only
n = 11
HDC+AuHCR
n = 1
