# Supplementary material for: Salvage therapies for first relapse of SHH medulloblastoma in early childhood
Source: Neuro Oncol. 2025 Apr 5;27(8):2158–69. doi: 10.1093/neuonc/noaf092 (PMC12448823; doi:10.1093/neuonc/noaf092)
Supplement: noaf092_suppl_Supplementary_Tables_S1-S2_Figures_S1-S4 [file noaf092_suppl_supplementary_tables_s1-s2_figures_s1-s4.zip › Supplemental Figure Legend.docx]

Supplemental Figure Legend

Supplemental Figure 1. Conventional chemotherapy regimen used upfront. Abbreviations CPM = cyclophosphamide, VCR = vincristine, VP16 = etoposide, CDDP = cisplatin, HD MTX = high-dose methotrexate, PCB = procarbazine

Supplemental Figure 2. Detailed radiation therapy management by pattern of relapse

Supplemental Figure 3. Detailed treatment combinations for low-dose salvage CSI patients

Supplemental Figure 4. Outcome comparison using Kaplan-Meier stratified by type of salvage regimen used.
