# Supplementary material for: Salvage therapies for first relapse of SHH medulloblastoma in early childhood
Source: Neuro Oncol. 2025 Apr 5;27(8):2158–69. doi: 10.1093/neuonc/noaf092 (PMC12448823; doi:10.1093/neuonc/noaf092)
Supplement: noaf092_suppl_Supplementary_Tables_S1-S2_Figures_S1-S4 [file noaf092_suppl_supplementary_tables_s1-s2_figures_s1-s4.zip › Supplemental Table 1 - molecularly defined.docx]

| Platform Type | n = 113 |  | | | n = 42 |
| --- | --- | --- | --- | --- | --- |
| Methylation array | 81 | |  | SHH 1/β = 25  SHH2/γ = 16 | |
| Immunohistochemistry | 11 | |  |  | |
| Nanostring | 8 | |  |  | |
| TaqMan low-density array | 3 | |  |  | |
| Next-generation sequencing | 3 | |  |  | |
| Single Nucleotide Polymorphism | 1 | |  |  | |
| Not documented | 6 | |  | SHH 1/β = 1 | |
