# Supplementary material for: Salvage therapies for first relapse of SHH medulloblastoma in early childhood
Source: Neuro Oncol. 2025 Apr 5;27(8):2158–69. doi: 10.1093/neuonc/noaf092 (PMC12448823; doi:10.1093/neuonc/noaf092)
Supplement: noaf092_suppl_Supplementary_Tables_S1-S2_Figures_S1-S4 [file noaf092_suppl_supplementary_tables_s1-s2_figures_s1-s4.zip › Supplemental Table 2 - SHH1v2.docx]

|  | SHH1/β (n = 26) | | SHH2/γ (n = 16) | | | p-value |
| --- | --- | --- | --- | --- | --- | --- |
| UPFRONT (Entire Cohort) | | | | | | |
| Gender (male) | | 18 | | 7 | 0.12 | |
| Diagnosis Era  1995-2006  2007-2017 | | 2  24 | | 1  15 | 1 | |
| Age original diagnosis  < 24 months | | 17 | | 3 | 0.005 | |
| Metastatic status  M0  M+ | | 16  9 | | 12  4 | 0.51 | |
| Histology  Classic  ND/MBEN  LCA | | 1  24  1 | | 1  15  0 | 1 | |
| HDC (Yes) | | 2 | | 0 | 0.52 | |
| RELAPSE (Entire Cohort) | | | | | | |
| Relapse timepoint  On therapy | | 10 | | 7 | 0.91 | |
| Time from diagnosis to relapse  < 12 months | | 15 | | 6 | 0.34 | |
| Relapse pattern  Local | | 12 | | 7 | 0.43 | |
| RELAPSE (Curative Intent Cohort) | | | | | | |
|  | | SHH1/β (n = 22) | | SHH2/γ (n = 15) | p-value | |
| Surgery (Yes) | | 2 | | 4 | 0.20 | |
| Radiation type  No RT  CSI  Focal RT only | | 6  12  4 | | 2  11  2 | 0.57 | |
| HDC (Yes) | | 2 | | 4 | 0.20 | |
| Alive (Yes) | | 12 | | 9 | 1 | |
