# Supplementary figures and images for: Salvage therapies for first relapse of SHH medulloblastoma in early childhood
Source: Neuro Oncol. 2025 Apr 5;27(8):2158–69. doi: 10.1093/neuonc/noaf092 (PMC12448823; doi:10.1093/neuonc/noaf092)

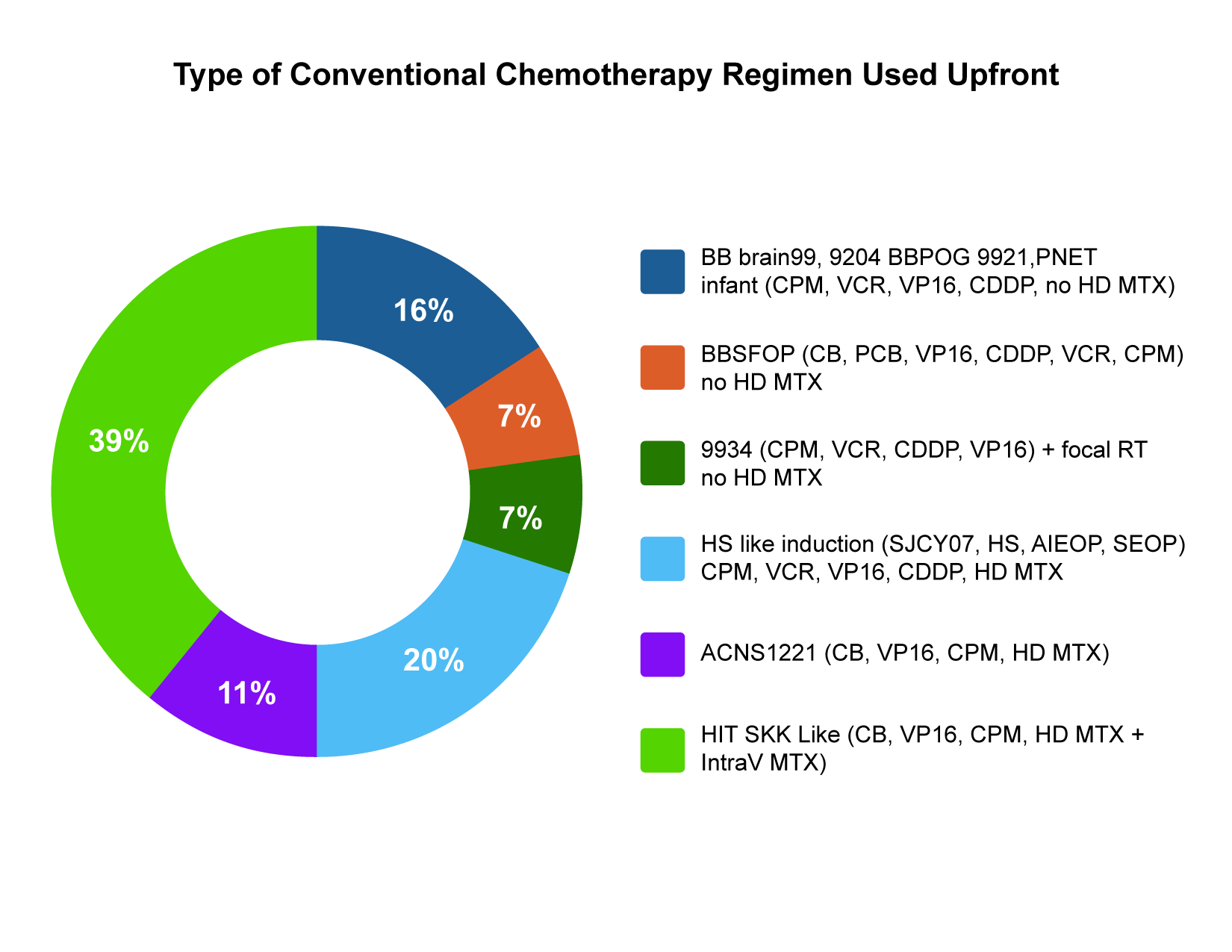

Supplement: noaf092_suppl_Supplementary_Tables_S1-S2_Figures_S1-S4 [file noaf092_suppl_supplementary_tables_s1-s2_figures_s1-s4.zip › Supplemental Figure 1. Type of Conventional Chemo Regimens.tif]

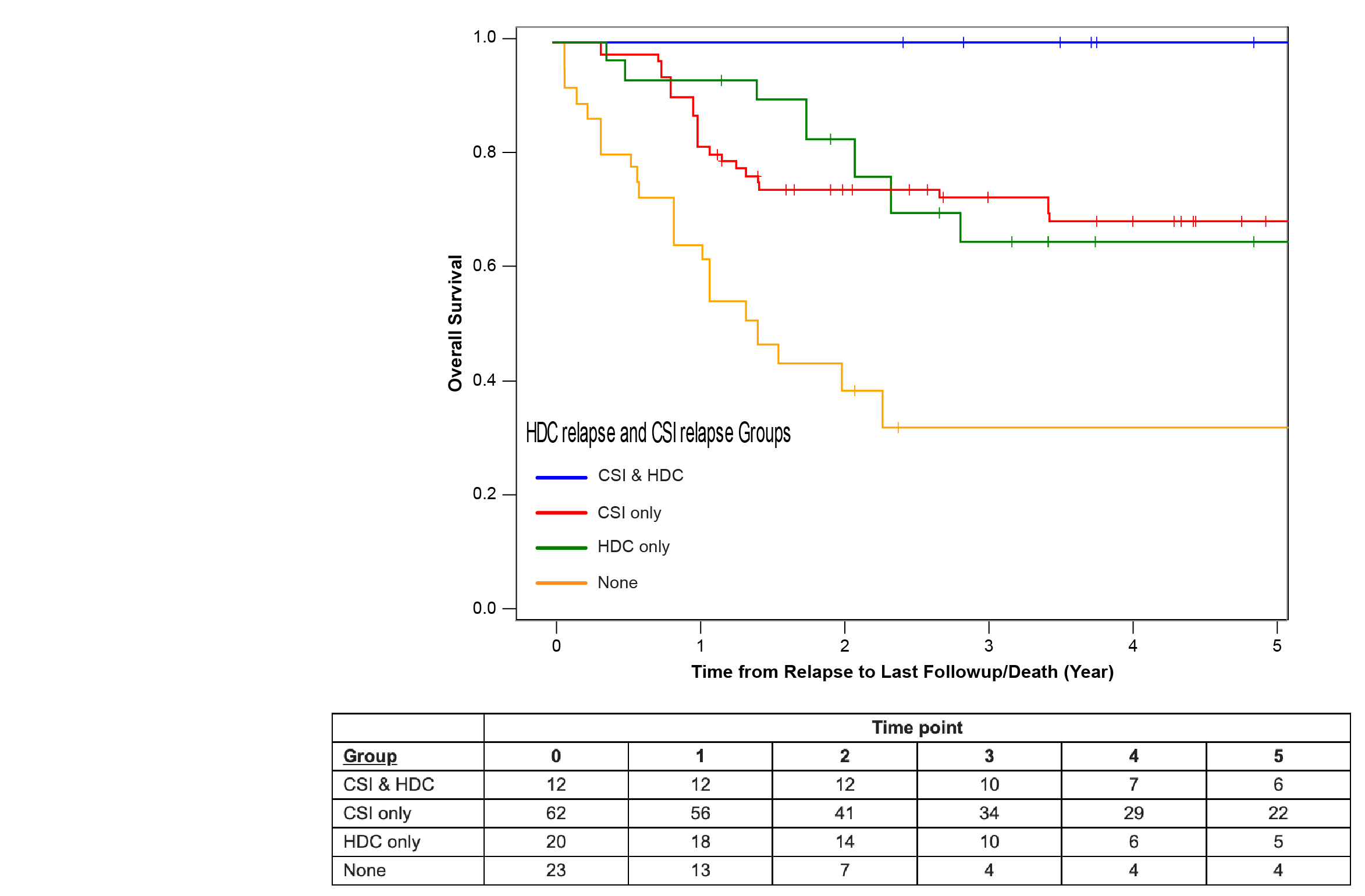

Supplement: noaf092_suppl_Supplementary_Tables_S1-S2_Figures_S1-S4 [file noaf092_suppl_supplementary_tables_s1-s2_figures_s1-s4.zip › Supplemental Figure 4. Salvage Therapy Approaches-EDIT.tif]
